# A comparative study to evaluate CT-based semantic and radiomic features in preoperative diagnosis of invasive pulmonary adenocarcinomas manifesting as subsolid nodules

**DOI:** 10.1038/s41598-020-79690-4

**Published:** 2021-01-18

**Authors:** Yun-Ju Wu, Yung-Chi Liu, Chien-Yang Liao, En-Kuei Tang, Fu-Zong Wu

**Affiliations:** 1 Section of Thoracic and Circulation Imaging, Department of Radiology, Kaohsiung Veterans General Hospital, Taiwan, No.386, Ta-Chung 1st Road, Kaohsiung, 81362 Taiwan; 2 Department of Radiology, Xiamen Chang Gung Hospital, Xiamen, China; 3 Department of Surgery, Kaohsiung Veterans General Hospital, Kaohsiung, Taiwan; 4 Department of Nursing, Shu-Zen Junior College of Medicine and Management, Kaohsiung, Taiwan; 5 Department of Medical Imaging and Radiology, Shu-Zen Junior College of Medicine and Management, Kaohsiung, Taiwan; 6 Faculty of Medicine, School of Medicine, National Yang Ming University, Taipei, Taiwan; 7 Institute of Clinical Medicine, National Yang Ming University, Taipei, Taiwan

**Keywords:** Cancer, Cancer, Diseases, Health care, Mathematics and computing

## Abstract

This study aims to predict the histological invasiveness of pulmonary adenocarcinoma spectrum manifesting with subsolid nodules ≦ 3 cm using the preoperative CT-based radiomic approach. A total of 186 patients with 203 SSNs confirmed with surgically pathologic proof were retrospectively reviewed from February 2016 to March 2020 for training cohort modeling. The validation cohort included 50 subjects with 57 SSNs confirmed with surgically pathologic proof from April 2020 to August 2020. CT-based radiomic features were extracted using an open-source software with 3D nodular volume segmentation manually. The association between CT-based conventional features/selected radiomic features and histological invasiveness of pulmonary adenocarcinoma status were analyzed. Diagnostic models were built using conventional CT features, selected radiomic CT features and experienced radiologists. In addition, we compared diagnostic performance between radiomic CT feature, conventional CT features and experienced radiologists. In the training cohort of 203 SSNs, there were 106 invasive lesions and 97 pre-invasive lesions. Logistic analysis identified that a selected radiomic feature named GLCM_Entropy_log10 was the predictor for histological invasiveness of pulmonary adenocarcinoma spectrum (OR: 38.081, 95% CI 2.735–530.309, *p* = 0.007). The sensitivity and specificity for predicting histological invasiveness of pulmonary adenocarcinoma spectrum using the cutoff value of CT-based radiomic parameter (GLCM_Entropy_log10) were 84.8% and 79.2% respectively (area under curve, 0.878). The diagnostic model of CT-based radiomic feature was compared to those of conventional CT feature (morphologic and quantitative) and three experienced radiologists. The diagnostic performance of radiomic feature was similar to those of the quantitative CT feature (nodular size and solid component, both lung and mediastinal window) in prediction invasive pulmonary adenocarcinoma (IPA). The AUC value of CT radiomic feature was higher than those of conventional CT morphologic feature and three experienced radiologists. The c-statistic of the training cohort model was 0.878 (95% CI 0.831–0.925) and 0.923 (0.854–0.991) in the validation cohort. Calibration was good in both cohorts. The diagnostic performance of CT-based radiomic feature is not inferior to solid component (lung and mediastinal window) and nodular size for predicting invasiveness. CT-based radiomic feature and nomogram could help to differentiate IPA lesions from preinvasive lesions in the both independent training and validation cohorts. The nomogram may help clinicians with decision making in the management of subsolid nodules.

## Introduction

With the introduction of low-dose lung computed tomography for lung cancer screening worldwide in more recent years, there is an increasing number of non-smoking related lung cancer manifesting with subsolid nodules in Asian population^1–6^. In 2011, the International Association for the Study of Lung Cancer (IASLC), the American Thoracic Society, and the European Respiratory Society introduced a novel classification system for lung adenocarcinoma spectrum lesions, dividing into pre-invasive lesions including atypical adenomatous hyperplasia (AAH) and adenocarcinoma in situ (AIS), minimally invasive (MIA) and invasive pulmonary adenocarcinoma (IPA)^7,8^. In recent years, several studies have investigated that generally adenocarcinoma spectrum lesions manifested as subsolid nodules (SSNs) have more indolent natural course, especially groundglass nodules (GGNs)^9–11^. However, the clinical behavior of these SSNs can be diverse^12^. Therefore it is important to correctly diagnose these indolent lesions from invasive pulmonary adenocarcinomas preoperatively.

The present study aims at investigating the diagnostic performance of clinical characteristics, conventional CT features, and radiomic CT feature to differentiate invasive lesions from preinvasive lesions in subjects with SSNs. In addition, we compared diagnostic performance between radiomic CT feature and experienced radiologists. Validation and calibration were conducted to evaluate the performance of the CT-based radiomic model in the training and validation cohorts.

## Material and method

### Study cohort

**Figure 1:**
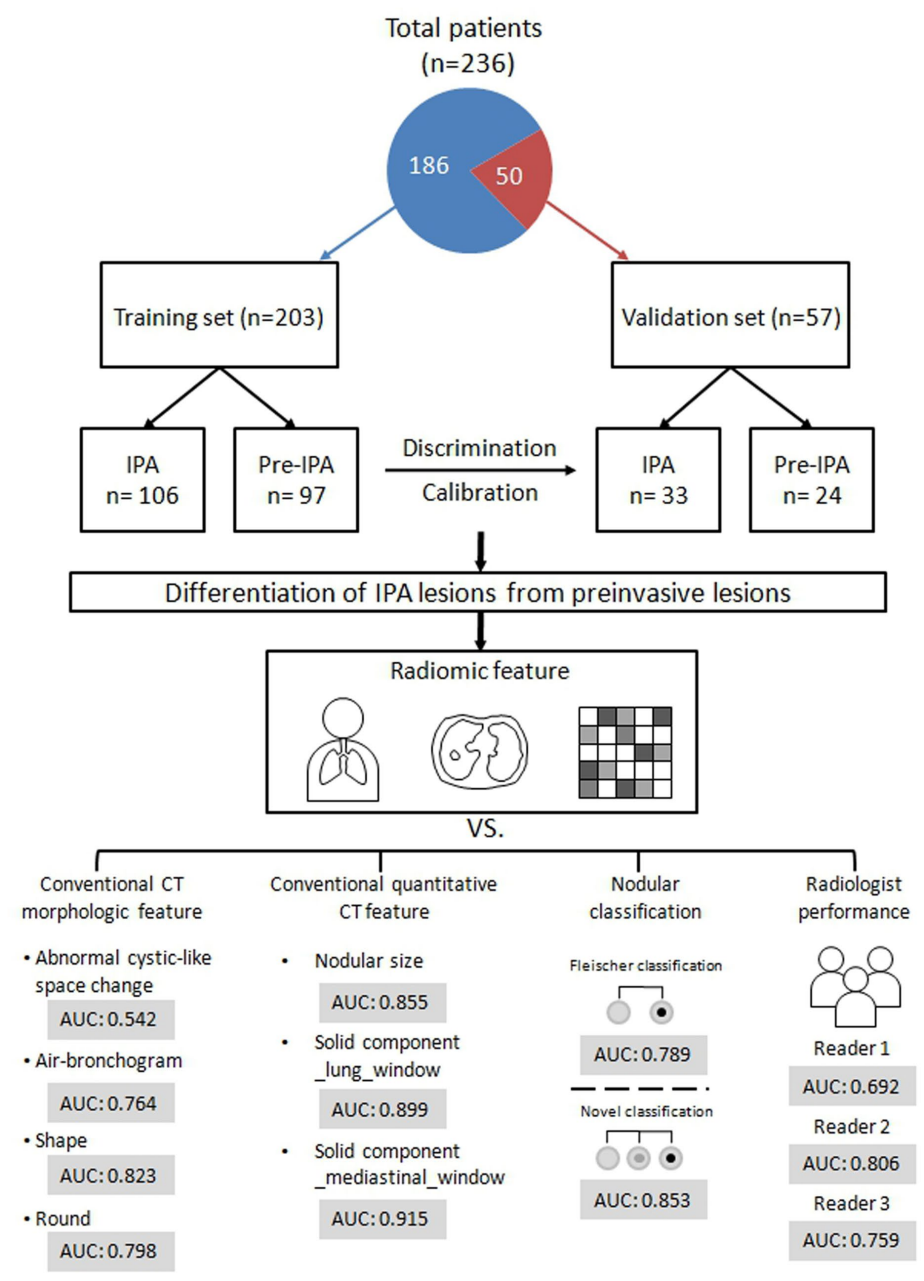
Overall study design flowchart for the training and validation cohorts and diagnostic performance by each approach.

The study population consisted of 186 subjects with 203 SSN pathologically proved and classified as pulmonary adenocarcinoma spectrum lesions according to the IASLC/ATS/ERS classification from February 2016 to March 2020 for training cohort modeling. The validation cohort included 50 subjects with 57 SSNs confirmed with surgically pathologic proof from April 2020 to August 2020. The flowchart summarizes the study design and diagnostic performance by each approach shown in Fig. 1. The inclusion criteria were as follows: (1) patients with SSNs ≦ 30 mm in diameter; (2) patients who did not receive preoperative treatment prior to surgery; (3) patients who underwent surgical resection within 3 months of CT; and (4) the pre-operative chest CT scan with thin-slice thickness before surgical intervention (≦ 2.5 mm). The protocol of this study was approved by the Institutional Review Board (IRB) of Kaohsiung Veterans General Hospital, and the study was followed the guidelines of the Helsinki Declaration. All methods were performed in accordance with the relevant guidelines and regulations. Written informed consent was waived due to the retrospective study design by the IRB of Kaohsiung Veterans General Hospital (No. VGHKS19-CT6-19).

### CT imaging protocol and acquisition

All preoperative chest CT scans were performed with a 16-slice CT (Somatom Sensation 16, Siemens Healthcare, Erlangen, Germany), a 64-slice CT (Aquilion 64; Toshiba Medical Systems), and 256-slice CT (Revolution CT, GE Healthcare, Milwaukee, USA) from the lung apex to the base without contrast enhancement as described in the previous study^13^. CT scans were acquired at full inspiration without contrast medium. The details of the scanning parameters using similar protocol for different vendors are listed as follows (Supplementary Table 1): Tube voltage, 120 kVp; body mass index (BMI)- dependent tube current 220 mAs to 350 mAs according to the BMI. Images were reconstructed with a section thickness of 1–2.5 mm using soft tissue kernel algorithm (different CT protocols in detail shown in Supplementary Table 1).

### Conventional CT features (qualitative and quantitative)

**Figure 2:**
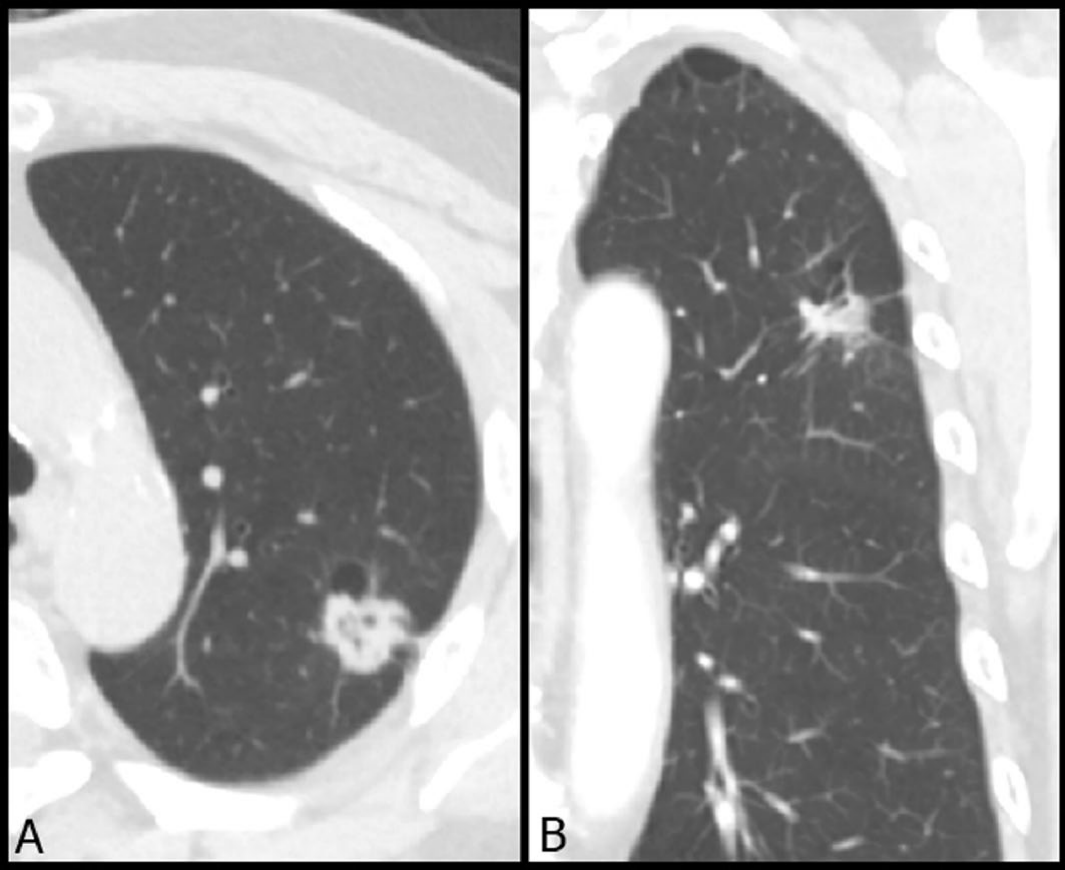
A typical example case of subsolid nodule with abnormal cystic-like airspace in LUL. A 65-year-old man had a 2.1 cm part-solid nodule with spiculated border in LUL. The (**A**) axial and (**B**) coronal CT images showed an abnormally dilated cystic-like airspace inside the lesion. The patient underwent video-thoracoscopic lobectomy of LUL. Further pathologic report demonstrated invasive pulmonary adenocarcinoma in LUL, Stage T1cN0M0. *LUL* left upper lobe.

**Figure 3:**
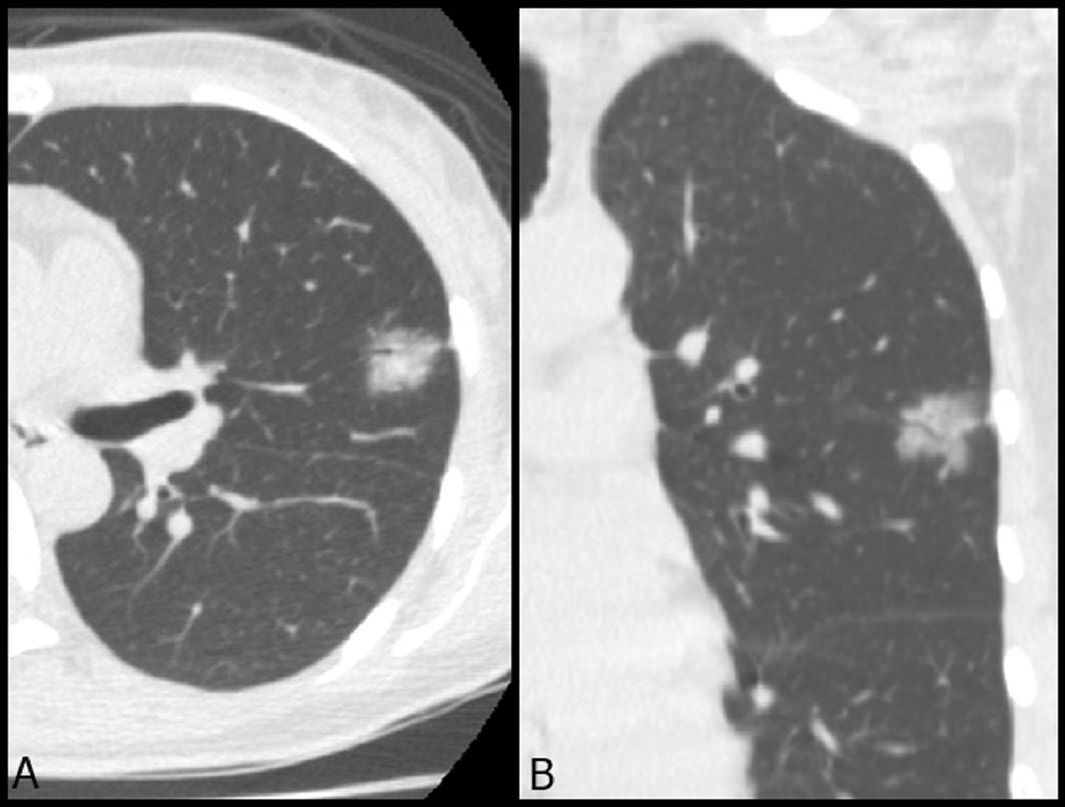
A typical example case of subsolid nodule with an air bronchogram sign in LUL. A 61-year-old woman had a 1.4 cm part-solid nodule in LUL. The (**A**) axial and (**B**) coronal images showed an internal air bronchogram inside the lesion. The patient underwent video-thoracoscopic wedge resection of LUL. Further pathologic report demonstrated invasive pulmonary adenocarcinoma in LUL, Stage T1bN0M0. *LUL* left upper lobe.

**Figure 4:**
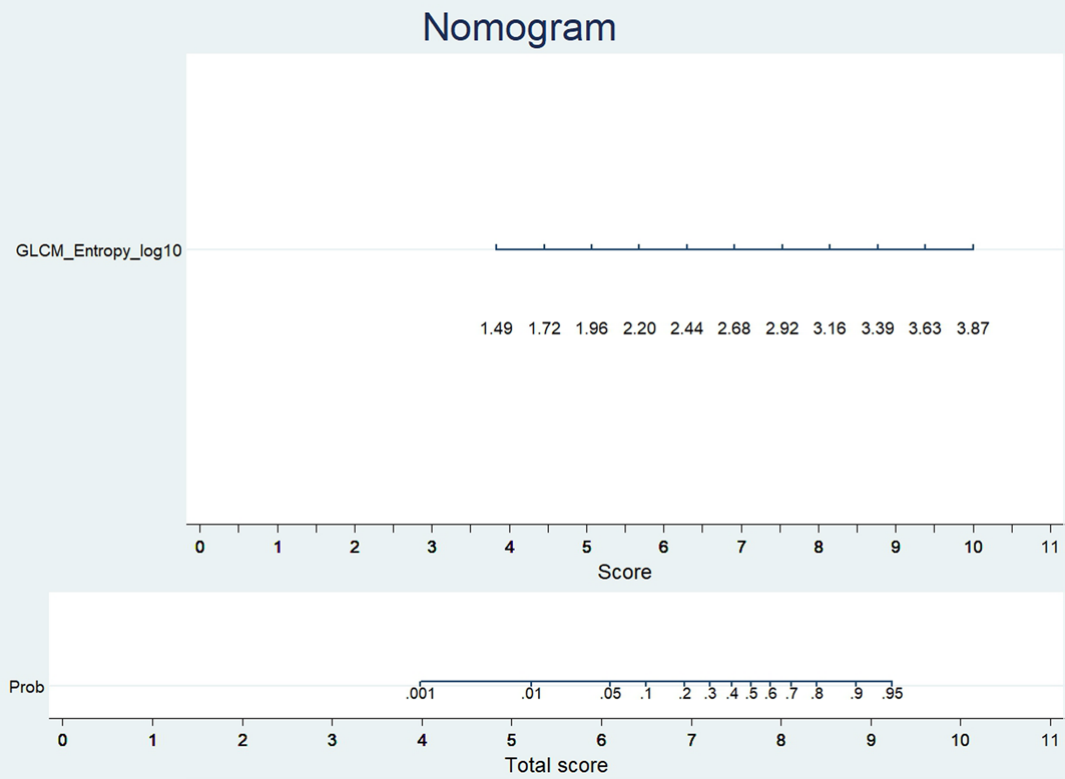
Nomogram to predict the possibility of invasive pulmonary adenocarcinoma lesions based on GLCM-based feature (GLCM_Entropy_log10). To use the nomogram, an individual participant’s value is located on each variable axis, and a line is drawn upward to determine the number of points received for each variable value. The sum of these numbers is located on the total points axis to determine the possibility of invasive pulmonary adenocarcinoma lesions.

**Figure 5:**
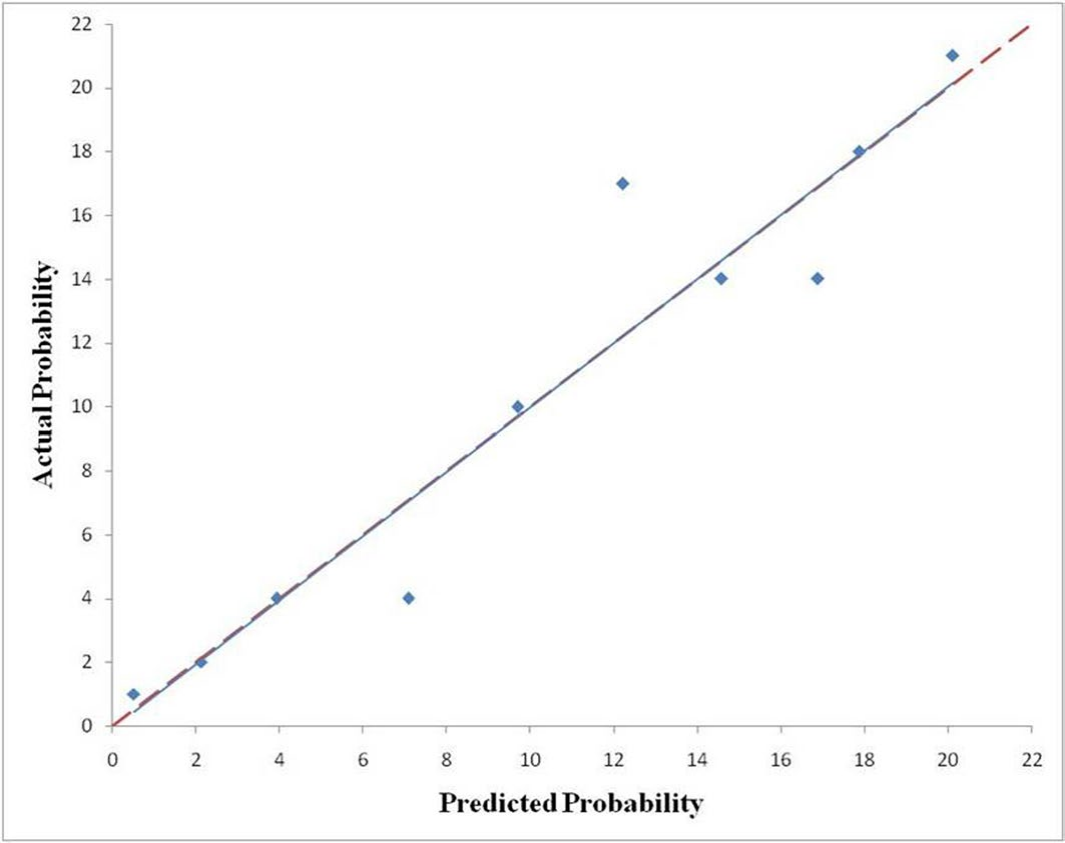
Calibration curves of the nomogram for predicting invasive pulmonary adenocarcinoma lesions from the training cohort. The Hosmer–Lemeshow test had a p value of 0.202 in the training cohort.

The assessments of radiologic characteristics were reviewed independently by two radiologists, who were blinded to the pathologic reports. Disagreements were solved in consensus. The CT-based features were based the following qualitative and quantitative data. Qualitative features were as the followings: (1) nodular type according to Fleischer classification (GGNs manifest as haziness opacity in the lung that does not obliterate the bronchovascular bundle; part-solid nodules consist of both ground-glass opacity and solid components)^14,15^; (2) novel nodular type according to the novel classification (classification into pure GGN, heterogeneous GGN (partly consolidated on lung windows), and part-solid nodules (with a mediastinal window solid component) according to the previous prospective study proposed by Kakinuma et al.)^10^; (3) abnormal cystic-like space change (an example shown in Fig. 2); (4) Air-bronchogram (an example shown in Fig. 3); (5) shape (smooth, lobulated or spiculated border); (6) round (oval or irregular). CT-based qualitative imaging features were recorded in consensus using long-axis diameter. Quantitative features were as the followings: (1) nodular size; (2) solid component in a mediastinal window; (3) solid component in a lung window. In addition, three readers were asked in the interpretation of each SSN according to 2 levels: preinvasive lesions or invasive lesions. A diagnostic performance comparison was conducted between radiomic CT feature and the three radiologists in the classification between preinvasive lesions and invasive lesions in the training cohort.

### Quantitative radiomic CT feature

Radiomic features of these 203 SSNs were extracted using the LifeX package (LifeX, version 5.10, Orsay, France, http://www.lifexsoft.org) for nodule segmentation with volume of interest (VOI) of at least 64 voxels for training cohort modeling^16^. The contours of these SSNs were delineated manually by an experienced thoracic radiologist. Regions of interest (ROI) were delineated around the nodule boundary for each section. A total of 41 features were derived from CT images and group according to intensity, shape, and second and higher-order features (Supplementary Table 2). For the histogram of the gray level distribution, the following features were extraction: the minimum, maximum, mean, and standard deviation of the Hounsfield units (HU) distribution. For first-order metrics extracted from the histogram, the following features were extraction: SkewnessH, KurtosisH, EntropyH and EnergyH. For second order metrics calculated from co-occurrence matrices: the following features were extraction: homogeneity, energy, contrast, correlation, entropy and dissimilarity. For higher-order metrics extracted from the grey-level histogram, the parameters included features of grey-level co-occurrence matrix (GLCM), neighborhood grey-level dependence matrix (NGLDM), grey-level run length matrix (GLRLM), and grey-level zone length matrix (GLZLM).

### Pathologic evaluation

All surgical resected specimens were fixed in 10% formalin and embedded in paraffin with haematoxylin and eosin staining for pathological diagnosis. The surgically resected SSNs specimens were histopathologically analyzed by two senior pathologists experienced in lung pathology classified as AAH, AIS, MIA, and IPA.

According to the revised lung adenocarcinoma (IASLC/ATS/ERS) classification of 2011^7,8^. The discordant cases were subsequently discussed in a consensus meeting until a consensus was obtained. All SSNs were divided into two groups: a preinvasive lesions group (AAH, AIS and MIA lesions) and invasive lesions group (invasive adenocarcinoma lesions) according to the revised lung adenocarcinoma (IASLC/ATS/ERS) classification.

### Interobserver agreement

To calculate the interobserver agreement of conventional CT feature, radiomic CT feature and radiologists, a random sample of 40 SSNs was investigated. The intraclass correlation coefficient (ICC) was used, and the ICC values were graded as follows: 0.0–0.20, slight; 0.21–0.40, fair; 0.41–0.60, moderate; 0.61–0.80, substantial; 0.81–1.00, almost perfect agreement.

### Statistical analyses

All statistical analyses were performed using SPSS 22.0 for Windows (SPSS Inc, Chicago, IL) and Stata version 13.1 (StataCorp, College Station, Texas 77845 USA). Because all the continuous variables are normally distributed, Student’s t-test was used to test the differences between two groups. Continuous variables are presented as mean ± standard deviation (SD). Categorical variables were summarized as frequencies and percentages and compared using the chi-square or Fisher exact test to examine differences in demographic characteristics. Univariate and multivariate logistic regression were used to determine these parameters for differentiating IPA lesions from preinvasive lesions. The results were expressed as an odds ratio (OR) with a 95% confidence interval (CI). Receiver operating characteristic (ROC) curve for the model was constructed, and the area under the curve (AUC) was calculated to compare the diagnostic performance of conventional CT features, radiomic CT feature and three experienced radiologists. In addition, sensitivity, specificity, PPV, NPV, positive LR (LR+) and negative LR (LR−) were calculated to measure the overall accuracy of the multiple tests. Calibration was assessed by the Hosmer–Lemeshow goodness-of-fit statistic and by calibration graphs plotting predicted IPA against the observed rates in deciles of predicted risk. A nomogram was established based on the radiomic parameter in the training cohort. The statistical significance for all tests was set at *P* < 0.05.

## Result

### Demographics and clinical characteristics

We retrospectively review thin-slice thickness images of 203 SSNs in 186 subjects who had subsolid nodule(s) preoperatively and subsequently underwent surgical resection with pathologically confirmed adenocarcinoma spectrum lesions at our hospital within the three-month interval for the training cohort modeling. Of the 203 SSNs, 97 SSNs had pre-invasive lesions and 106 SSNs had invasive lesions.

Table 1 summarizes the patients’ characteristics in the training and validation cohorts.

**Table 1:** Patients’ demographic and clinical characteristics in the training and validation cohorts.

	Training cohort (n = 203)	Validation cohort (n = 57)	P
**Gender (n, %)**			0.107
Male	58 (28.6%)	10 (17.9%)	
Female	145 (71.4%)	46 (82.1%)	
Age (year, n, %)	59.33 ± 9.45	60.54 ± 10.09	0.399
**Smoking history (n, %)**			0.511
No	177 (87.2%)	8 (80.0%)	
Yes	26 (12.8%)	2 (20.0%)	
**Lesion location (n, %)**			0.966
Right upper lobe	67 (33.0%)	20 (35.1%)	
Right middle lobe	13 (6.4%)	4 (7.0%)	
Right lower lobe	41 (20.2%)	13 (22.8%)	
Left upper lobe	55 (27.1%)	13 (22.8%)	
Left lower lobe	27 (13.3%)	7 (12.3%)	
**Nodular type (Fleischer classification, n, %)**			0.124
GGN	68 (33.5%)	13 (22.8%)	
PSN	135 (66.5%)	44 (77.2%)	
**Nodular type (Novel classification, n, %)**			0.063
GGN	68 (33.5%)	13 (22.8%)	
Heterogeneous GGN	19 (9.4%)	2 (3.5%)	
PSN	116 (57.1%)	42 (73.7%)	
Nodular size	1.639 ± 1.029	1.629 ± 1.048	0.950
Solid part_lung_window	0.728 ± 0.838	0.874 ± 0.827	0.289
Solid part_mediastinal_window	0.559 ± 0.849	0.683 ± 0.921	0.340
**Cystic change (n, %)**			0.098
0	187 (92.1%)	56 (98.2%)	
1	16 (7.9%)	1 (1.8%)	
**Airbronchogram (n, %)**			0.402
0	112 (55.2%)	35 (61.4%)	
1	91 (44.8%)	22 (38.6%)	
**Shape (n, %)**			0.868
Smooth	61 (30.0%)	19 (33.3%)	
Lobulated	98 (48.3%)	27 (47.4%)	
Spiculated	44 (21.7%)	11 (19.3%)	
**Round (n, %)**			0.130
Oval	84 (41.4%)	30 (52.6%)	
Irregular	119 (58.6%)	27 (47.4%)	

*GGN* groundglass nodule, *PSN* part-solid nodule.

For clinical characteristics, there were no significant differences in the percentage of sex ratio, smoking history, lesion location, cystic change, airbronchogram, shape, and round between these two groups. Compared with the validation cohort, there were no differences in age, nodular size, solid component_lung_window, and solid component_mediastinal_window in the training cohort shown in Table 1.

In the selected 12 features in this study cohort, there were no significant differences in the training cohort and validation cohort in terms of CONVENTIONAL_HUmean, CONVENTIONAL_HUstd, CONVENTIONAL_HUQ2, CONVENTIONAL_HUQ3, HISTO_Entropy_log10, HISTO_Entropy_log2, GLCM_Entropy_log10, GLCM_Entropy_log2 (= Joint entropy), GLRLM_HGRE, GLRLM_SRHGE, GLZLM_HGZE, GLZLM_SZHGE shown in Table 2. Univariate and multiple logistic regression analyses of conventional CT characteristics and radiomic texture features in prediction of invasive lesions are shown in Table 3. The results of the univariate logistic regression model suggested that all conventional CT characteristics and radiomic texture features had significant association on the prediction of invasive lesions. Based on multiple logistic regression analyses, GLCM_Entropy_log10 was the only one independently important predictor for invasive lesions.

**Table 2:** Selected radiomic features of the study population with SSNs in the training and validation cohorts.

	Training cohort (n = 203)	Validation cohort (n = 57)	P
CONVENTIONAL_HUmean	− 510.667 ± 163.021	− 484.854 ± 158.844	0.290
CONVENTIONAL_HUstd	175.195 ± 67.379	180.957 ± 64.327	0.565
CONVENTIONAL_HUQ2	− 521.009 ± 176.907	− 492.524 ± 182.305	0.287
CONVENTIONAL_HUQ3	− 385.738 ± 217.710	− 355.428 ± 210.364	0.351
HISTO_Entropy_log10	1.757 ± 0.187	1.775 ± 0.159	0.516
HISTO_Entropy_log2	5.837 ± 0.621	5.895 ± 0.529	0.516
GLCM_Entropy_log10	2.974 ± 0.436	2.997 ± 0.423	0.727
GLCM_Entropy_log2 (= Joint entropy)	9.880 ± 1.450	9.956 ± 1.404	0.727
GLRLM_HGRE	3047.072 ± 1936.843	3302.469 ± 1898.093	0.378
GLRLM_SRHGE	2960.436 ± 1832.796	3208.952 ± 1825.607	0.367
GLZLM_HGZE	2909.645 ± 1736.698	3146.723 ± 1668.209	0.360
GLZLM_SZHGE	2312.917 ± 1352.563	2502.086 ± 1393.449	0.355

*HU* hounsfield unit, *GLCM* gray-level co-occurrence matrix, *GLRLM* grey-level run length matrix, *HGRE* high grey-level run emphasis; SRHGE: short-run high grey-level emphasis, *GLZLM* grey-level zone length matrix, *HGZE* high grey-level zone emphasis, *SZHGE* short-zone high grey-level emphasis.

**Table 3:** Univariate and multivariate logistic regression model to differentiate invasive lesions from preinvasive lesions.

Characteristic	Univariate logistic regression		Multivariate logistic regression	
	OR (95% CI)	P	OR (95% CI)	P
CONVENTIONAL_HUmean	1.016 (1.012–1.021)	< 0.001	0.998 (0.984–1.013)	0.835
GLCM_Entropy_log10	127.825 (33.639–485.725)	< 0.001	38.081 (2.735–530.309)	0.007
GLZLM_SZHGE	1.002 (1.002–1.003)	< 0.001	1.002 (1.000–1.004)	0.061
Nodular size	5.119 (3.039–8.623)	< 0.001	1.017 (0.330–3.134)	0.977
Solid component_lung_window	28.368 (11.126–72.328)	< 0.001	1.474 (0.159–13.688)	0.733
Solid component_mediastinal_window	452.340 (56.553–3618.028)	< 0.001	0.925 (0.126–6.764)	0.938
Nodular type (Fleischer classification)	29.524 (11.740–74.244)	< 0.001	1.928 (0.309–12.008)	0.482
Nodular type (Novel classification)	11.233 (5.896–21.400)	< 0.001	0.826 (0.281–2.433)	0.729
Abnormal cystic-like space change	4.380 (1.208–15.875)	0.025	0.840 (0.088–8.034)	0.880
Air-bronchogram	10.882 (5.581–21.220)	< 0.001	0.588 (0.161–2.143)	0.421
Shape	11.918 (5.896–24.091)	< 0.001	0.823 (0.202–3.363)	0.787
Round	17.037 (8.322–34.877)	< 0.001	3.068 (0.765–12.307)	0.114

*HU* hounsfield unit, *GLCM* gray-level co-occurrence matrix, *OR* odds ratio, *GLZLM* grey-level zone length matrix, *SZHGE* short-zone high grey-level emphasis.

Table 4 shows the sensitivity, specificity, negative predictive value (NPV), positive predictive value (PPV), likelihood ratio (LR) (+), and LR (−) values based on conventional CT features and radiomic features for invasive lesions prediction with SSNs. A comparison of diagnostic performance of conventional CT feature, radiomic texture features and three radiologists in prediction of invasive lesions are summarized in Table 5. Diagnostic performance showed that GLCM_Entropy_log10 was the best predictor for differentiating preinvasive lesions from invasive lesions. The optimal cut-off value for GLCM_Entropy_log10 in differentiating preinvasive lesions from invasive lesions was with a sensitivity of 84.80% and a specificity of 79.20% (PPV = 81.66%; NPV = 82.66%). In model 1, GLCM_Entropy_log10 had the largest AUC value of 0.878, which was significantly higher than those of the conventional CT morphologic characteristics (abnormal cystic-like space change: 0.542; air-bronchogram: 0.764; shape: 0.823; round: 0.798). In the model 2, GLCM_Entropy_log10 had the similar diagnostic performance with conventional quantitative CT features. Among these potential quantitative CT features predictive parameters, nodule size was the most sensitive sign. However, the solid components (mediastinal and lung window) were the two parameters with optimal balance between the sensitivity and specificity. To compare with diagnostic performance of radiomic features versus subsolid nodule’s classification system (Fleischer and novel classification system), the model 3 showed that GLCM_Entropy_log10 had the similar diagnostic performance with the novel SSN classification system. However, GLCM_Entropy_log10 had superior diagnostic performance over the Fleischer classification system in invasion lesion’s prediction.

**Table 4:** The diagnostic performance based on conventional CT features and radiomic features for invasive lesions prediction with SSNs.

Algorithm model	Cut-off	AUC (95% CI)	Sensitivity	Specificity	Positive LR	Negative LR	PPV %	NPV %
CONVENTIONAL_HUmean	− 519.7558	0.910 (0.862–0.946)	75.2	89.6	7.22	0.28	88.76	76.78
GLCM_Entropy_log10	2.963	0.878 (0.831–0.925)	84.8	79.2	4.07	0.19	81.66	82.66
GLZLM_SZHGE	2186.5562	0.922 (0.876–0.955)	80.0	89.6	7.68	0.22	89.37	80.39
Nodular size	1.04	0.852 (0.796–0.898)	94.3	64.9	2.69	0.087	74.59	91.25
Solid component_lung_window	0.4	0.900 (0.851–0.938)	90.6	84.5	5.86	0.11	86.46	89.16
Solid component_mediastinal_window	0.09	0.916 (0.869–0.950)	89.6	84.5	5.80	0.12	86.33	88.15
Nodular type (Fleischer classification)	PSN	0.791 (0.729–0.845)	94.3	63.9	2.61	0.089	74.05	91.12
Nodular type (Novel classification)	PSN	0.854 (0.798–0.900)	91.5	77.3	4.03	0.11	81.49	89.28
Abnormal cystic-like space change	(+)	0.546 (0.475–0.616)	12.3	96.9	3.97	0.91	81.25	50.28
Air-bronchogram	(+)	0.761 (0.697–0.818)	69.8	82.5	3.98	0.37	81.33	71.43
Shape	spiculated	0.823 (0.763–0.873)	92.5	54.6	39.35	0.60	69.00	86.95
Round	Irregular	0.795 (0.733–0.848)	86.8	72.2	3.12	0.18	77.33	83.35

*SSN* subsolid nodule, *AUC* area under curve, *HU* hounsfield unit, *GLCM* gray-level co-occurrence matrix, *GLZLM* grey-level zone length matrix, *SZHGE* short-zone high grey-level emphasis, *PSN* part-solid nodule.

**Table 5:** Comparison of ROC curves for radiomic feature, conventional CT feature and radiologists in differential diagnosis of invasive lesions versus preinvasive lesions.

	AUC (%)	Sensitivity (%)	Specificity (%)	P
**Model 1: Conventional CT morphologic feature**				
GLCM_Entropy_log10 (reference)	0.878	84.8	79.2	
Abnormal cystic-like space change	0.542	12.3	96.9	< 0.001
Air-bronchogram	0.764	69.8	82.5	< 0.001
Shape	0.823	92.5	54.6	0.049
Round	0.798	86.8	72.2	0.008
**Model 2 Conventional quantitative CT feature**				
GLCM_Entropy_log10 (reference)	0.878	84.8	79.2	
Nodular size	0.855	94.3	64.9	0.319
Solid component_lung_window	0.899	90.6	84.5	0.385
Solid component_mediastinal_window	0.915	89.6	84.5	0.104
**Model 3 Nodular classification**				
GLCM_Entropy_log10 (reference)	0.878	84.8	79.2	
Nodular type (Fleischer classification)	0.789	94.3	63.9	0.002
Nodular type (Novel classification)	0.853	91.5	77.3	0.318
**Model 4 Radiologist performance**				
GLCM_Entropy_log10 (reference)				
Reader 1	0.692	41.0	97.9	< 0.001
Reader 2	0.806	78.3	82.5	< 0.001
Reader 3	0.759	52.8	98.9	< 0.001
All readers	0.753	57.4	93.1	< 0.001

*ROC* receiver operating characteristic, *AUC* area under curve, *GLCM* gray-level co-occurrence matrix.

In the model 4, GLCM_Entropy_log10 had the highest AUC value of 0.878, which was significantly higher than the AUC of the three experienced radiologists (radiologist 1: 0.692; radiologist 2: 0.806; radiologist 3: 0.759).

### Validation and calibration of the GLCM-based model and nomogram

**Figure 6:**
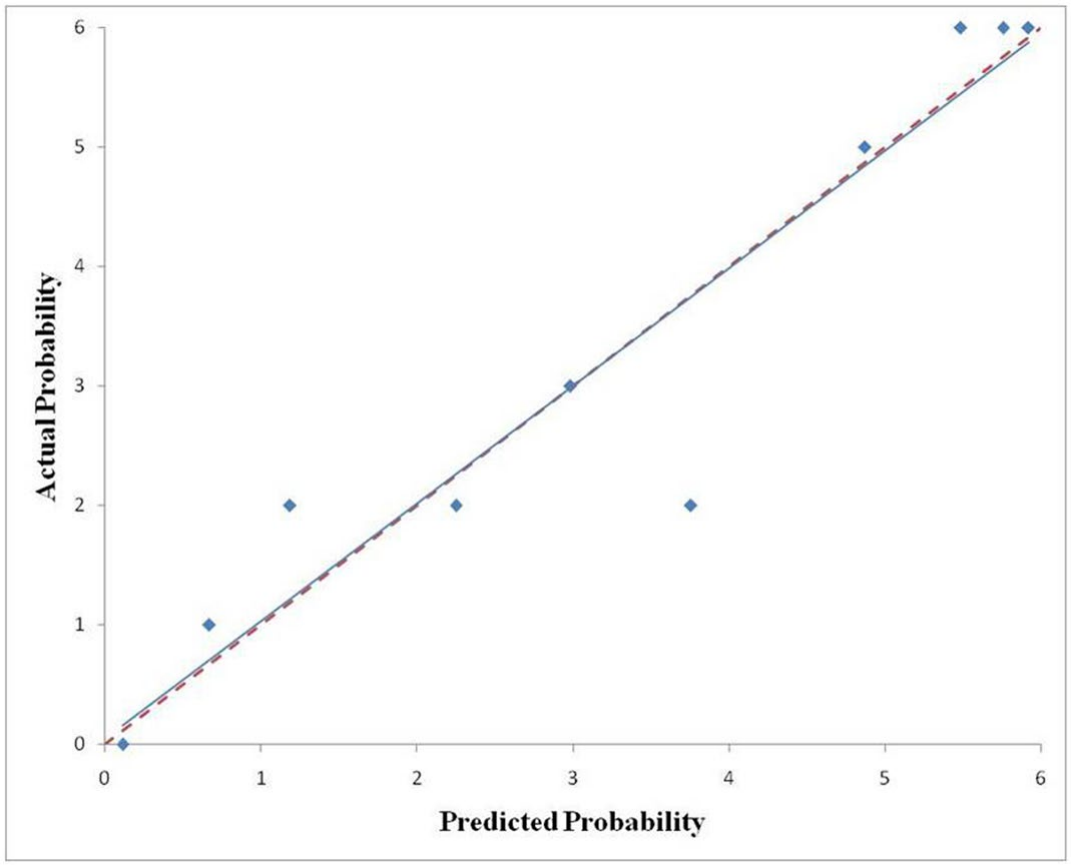
Calibration curves of the nomogram for predicting invasive pulmonary adenocarcinoma lesions from the validation cohort. The Hosmer–Lemeshow test had a p value of 0.917 in the validation cohort.

The GLCM-based (GLCM_Entropy_log10) radiomic model was then evaluated in the training and validation cohorts to assess the performance of discrimination and calibration with the Hosmer–Lemeshow goodness-of-fit test. A nomogram was constructed based on the regression model by the modeling strategies package in the Stata 13.1 software. We finally selected the GLCM-based feature (GLCM_Entropy_log10) to develop the radiomic nomogram in predicting IPA shown in Fig. 4. A GLCM-based (GLCM_Entropy_log10) radiomic nomogram showed good discrimination and goodness‐of‐fit for the training cohort (area under the receiver operating characteristic curve: 0.878 [95% CI, 0.831–0.925]; Hosmer–Lemeshow test, *P* = 0.202; calibration plot Fig. 5) and validation cohort (area under the receiver operating characteristic curve: 0.923 [95% CI, 0.854–0.991]; Hosmer‐Lemeshow test, *P* = 0.917; calibration plot Fig. 6).

### Interobserver agreement

The ICC was as follows: for 12 selected radiomic features, the range of ICC of all parameters was 0.978–0.994. For qualitative CT feature, the range of ICC of all parameters was 0.660–0.765. For quantitative CT feature, the range of ICC of all parameters was 0.903–0.959. For pulmonary adenocarcinoma spectrum classification, the range of ICC of three readers was 0.729–0.803.

## Discussion

The heterogeneous behaviors of persistent subsolid nodules are most frequently encountered diagnostic and management dilemmas in the Asian lung cancer screening program with high prevalence of non-smoking related lung cancers^3,4,13,17,18^. In addition, discrepancies in subsolid nodule categorization caused by disagreement on presence of a solid component, which may lead to different clinical decision and management^19–21^. In this context, the texture analysis of subsolid nodules has been recognized in differentiating invasive pulmonary adenocarcinomas from preinvasive lesions by quantitative assessment. To distinguish invasive pulmonary adenocarcinomas from preinvasive lesions is important in clinical decision making for lung cancer screening and subsolid nodule’s management^13,22,23^. In this study, our study results demonstrated that GLCM-based feature (GLCM_Entropy_log10) was the independent predictor for invasive pulmonary adenocarcinomas prediction.

We built a nomogram based on the GLCM-based feature (GLCM_Entropy_log10) to predict IPA, and it showed good discrimination and goodness-of-fit.

Furthermore, our study results demonstrate the superior performance of the GLCM-based feature (GLCM_Entropy_log10) over CT-based morphologic features in the study. The GLCM-based feature (GLCM_Entropy_log10) yielded a significantly higher AUC for prediction of invasive pulmonary adenocarcinomas when compared to the CT-based morphologic features. Previous studies have demonstrated that the solid component is the major determinant in prediction of invasive degree of the lung adenocarcinoma spectrum lesions^24–26^. These results are in line with our above findings. In addition, our study result demonstrated that GLCM-based feature (GLCM_Entropy_log10) has similar diagnostic performance to solid component (mediastinal window or lung window) in prediction of invasive lesions. In contrast to computer-aid texture quantitative analysis, CT-based quantitative and qualitative features perceived by naked eye will lead to a large inter-observer variability depended on radiologists^27^. In addition, imaging interpretation by the visual process through the naked eye could not fully understand the underlying biological heterogeneity of subsolid nodules. These findings suggest that texture analysis as a non-invasive, mathematical quantitative method of assessing that biological heterogeneity within the subsolid nodules might be of clinical relevance in predicting the pathologic invasiveness of the lesions of the pulmonary adenocarcinoma spectrums.

Previous studies have utilized different models of radiomic score to distinguish invasive pulmonary adenocarcinomas from preinvasive lesions that present as subsolid nodules ≦ 3 cm^28–32^. However different models with several different extracted radiomic features are utilized^33–35^. Therefore, the verification of research results is difficult to apply in the real world due to complex radio-score models. In the present study, we use a single simplified approach of the radiomic feature parameter in identifying the pathologic invasiveness of lung adenocarcinoma lesions and comparison with the performance of the conventional CT morphologic features and experienced radiologists. To the authors’ knowledge, no published studies have comprehensively investigated the difference of the diagnostic performance between the simplified radiomic parameter, conventional CT features and radiologists. In this model established with only one simplified texture feature generated for this study, the sensitivity, specificity, and AUC were 84.8%, 79.2% and 0.878 (95% CI 0.831–0.925), respectively. There was significant difference (abnormal cystic-like space change, p < 0.001; air-bronchogram, p < 0.001; shape, p = 0.049; round, p = 0.008) in the AUC between the models based on only one simplified texture feature and conventional CT morphologic features. In addition, the diagnostic performance of our model with only one simplified texture feature was higher than those of all three radiologists (all three readers, p < 0.001). In this study, our study result is in line with high intra-tumor heterogeneity associated with high entropy, suggestive of progression and invasiveness degree of adenocarcinoma spectrum lesions. Previous studies have demonstrated that histogram-based 75th–90th percentile CT numbers and entropy were best predictors to distinguish between IPA and AIS-MIA^36^. In addition, we identify only one simplified second-order GLCM-based quantitative statistical texture parameter which represents the whole-tumor texture feature to significantly differentiate invasive lesions from preinvasive lesions. In this study, the manual segmentation of SSNs usually takes 3 min delineated in a dozen of slices.

In the future, a deep-learning based automatic nodule segmentation can be used to extract this specific GLCM-based feature, and therefore to develop a computer-aided detection system to assist clinical decision-making in differentiation IPA lesions from preinvasive lesions.

The main strength of this study is that we established a simplified radiomic signature based on only one-second order statistical radiomic feature, which showed better diagnostic performance in differentiation of IPA from pre-invasive lesions compared with those of conventional CT morphologic model or experienced three radiologists.

In addition, GLCM-based feature (GLCM_Entropy_log10) has similar diagnostic performance to solid component (mediastinal window or lung window) in prediction of invasive lesions. However, our study has several limitations. First, there as a potential of patient selection bias due to the retrospective single-site study. Further validation of these results in prospective multi-center studies is warranted. Second, nodule segmentation was performed manually by experienced radiologists, which may significantly contribute to interobserver variability^27^. However, the results of interobserver variability was very low according to our preliminary report based on 40 cases. Third, different CT vendors with lack of standardization of scanning parameters would limit the external validity and generalizability of study results in the real-world practice^37–40^. However, all the study subjects in our study were performed with thin slice thickness of ≦ 2.5 mm that had met ACR accreditation for LDCT imaging protocols.

## Conclusion

In conclusion, a simplified radiomic signature and nomogram based on GLCM-based feature (GLCM_Entropy_log10) could help to differentiate invasive lesions from pre-invasive lesions groups. For invasive lesion’s prediction, the value of GLCM-based feature (GLCM_Entropy_log10) higher than 2.963 yielded the optimal discrimination between invasive and preinvasive lesions groups, with a sensitivity and specificity of 84.8% and 79.2%, respectively. In addition, radiomic feature may provide superior diagnostic performance compared with those of morphologic CT features and radiologists. The nomogram may help clinicians with decision making in the management of subsolid nodules.

## Supplementary Information

Supplementary Information.

## Data Availability

The datasets used and/or analyzed during the current study are available from the corresponding author on reasonable request.
