# Supplementary material for: A comparative study to evaluate CT-based semantic and radiomic features in preoperative diagnosis of invasive pulmonary adenocarcinomas manifesting as subsolid nodules
Source: Sci Rep. 2021 Jan 18;11:66. doi: 10.1038/s41598-020-79690-4 (PMC7814025; doi:10.1038/s41598-020-79690-4)
Supplement: Supplementary file 1 — Supplementary Information. [file 41598_2020_79690_MOESM1_ESM.docx]

**Title Page**

**Title**

A comparative study to evaluate CT-based semantic and radiomic features in preoperative diagnosis of invasive pulmonary adenocarcinomas manifesting as subsolid nodules

**Authors:**

Yun-Ju Wu^1^^,+^, Yung-Chi Liu^2,+^, Chien-Yang Liao^1,+^, En-Kuei Tang^3,4^, Fu-Zong Wu^1,4,5,6^

**Author Affiliations:**

^1^Department of Radiology, Kaohsiung Veterans General Hospital, Kaohsiung, Taiwan

^2^Department of Radiology, Xiamen Chang Gung Hospital, China

^3^Department of Surgery, Kaohsiung Veterans General Hospital, Kaohsiung, Taiwan

^4^Department of Nursing, Shu-Zen Junior College of Medicine and Management, Kaohsiung, Taiwan

^5^Department of Medical Imaging and Radiology, Shu-Zen Junior College of Medicine and Management, Kaohsiung, Taiwan

^6^Faculty of Medicine, School of Medicine, National Yang Ming University, Taipei, Taiwan

^6^Institute of Clinical Medicine, National Yang Ming University, Taipei, Taiwan

^+^ Yun-Ju Wu, Liu Yung-Chi and Chien-Yang, Liao contributed equally to first authors.

Correspondence to:

* Fu-Zong, Wu, MD, Section of Thoracic and Circulation Imaging Department of Radiology, Kaohsiung Veterans General Hospital, Taiwan No.386, Ta-Chung 1st Road, Kaohsiung, Taiwan 81362; Faculty of Medicine, School of Medicine, National Yang Ming University, Taipei, Taiwan; Department of Medical Imaging and Radiology, Shu-Zen Junior College of Medicine and Management, Kaohsiung, Taiwan. E-mail contact: cmvwu1029@gmail.com

| Supplement Table 1. CT acquisition protocols and image reconstruction parameters | | | |
| --- | --- | --- | --- |
| Parameter | GE Revolution | Siemens Sensation 16 | Toshiba Aquilion 64 |
| Scan number (patient-based, Training set) | 84 | 12 | 90 |
| Scan number (patient-based, Validation set) | 31 | 2 | 17 |
| Acquisition mode | Helical | Helical | Helical |
| Tube voltage, kv | 120 | 120 | 120 |
| Tube current, mAs | 220-350 (smart mode) | 220-350 | 220-350 |
| Collimation, mm | 256 x 0.625 mm | 16 x 0.75 mm | 64 x 0.5 mm |
| Reconstruction kernel | Standard | Standard | FC08 |
| Slice thickness, mm | 1-2.5 mm | 1-2.5 mm | 1-2.5 mm |

| Supplement Table 2. LifeX radiomic feature analysis | | |
| --- | --- | --- |
| Feature name | | Abbreviation |
| Conventional features | |  |
|  | Minimum | CONVENTIONAL_HUmin |
|  | Mean | CONVENTIONAL_HUmean |
|  | Standard-deviation | CONVENTIONAL_HUstd |
|  | Maximum | CONVENTIONAL_HUmax |
|  | Quarter 1 | CONVENTIONAL_HUQ1 |
|  | Quarter 2 | CONVENTIONAL_HUQ2 |
|  | Quarter 3 | CONVENTIONAL_HUQ3 |
| Histogram features | |  |
|  | Skewness | HISTO_Skewness |
|  | Kurtosis | HISTO_Kurtosis |
|  | Excess of Kurtosis | HISTO_ExcessKurtosis |
|  | Entropy_log10 | HISTO_Entropy_log10 |
|  | Entropy_log2 | HISTO_Entropy_log2 |
|  | Energy | HISTO_Energy (=Uniformity) |
| SHAPE | |  |
|  | Volume (mL) | SHAPE_Volume (mL) |
|  | Volume (# vx) | SHAPE_Volume (# vx) |
|  | Sphericity (only for 3D ROI (nz >1)) | SHAPE_Sphericity (only for 3D ROI (nz>1) |
|  | Compacity (only for 3D ROI (nz >1)) | SHAPE_Compacity only for 3D ROI (nz>1) |
| Textural features based on the Gray-level Co-occurrence Matrix (GLCM) | | |
|  | Homogeneity | GLCM_Homogeneity (=Inverse difference) |
|  | Energy | GLCM_Energy (=Angular second moment) |
|  | Contrast | GLCM_Contrast (=Variance) |
|  | Correlation | GLCM_Correlation |
|  | Entropy_log10 | GLCM_Entropy_log10 |
|  | Entropy_log2 | GLCM_Entropy_log2 (=Joint entropy) |
|  | Dissimilarity | GLCM_Dissimilarity |
| Textural features based on the Gray-Level Run Length Matrix (GLRLM) | | |
|  | Short-Run Emphasis (SRE) | GLRLM_SRE |
|  | Long-Run Emphasis (LRE) | GLRLM_LRE |
|  | Low Gray-level Run Emphasis (LGRE) | GLRLM_LGRE |
|  | High Gray-level Run Emphasis (HGRE) | GLRLM_HGRE |
|  | Short-Run Low Gray-level Emphasis (SRLGE) | GLRLM_SRLGE |
|  | Short-Run High Gray-level Emphasis (SRHGE) | GLRLM_SRHGE |
|  | Long-Run Low Gray-level Emphasis (LRLGE) | GLRLM_LRLGE |
|  | Long-Run High Gray-level Emphasis (LRHGE) | GLRLM_LRHGE |
|  | Gray-level Non-Uniformity (GLNU) | GLRLM_GLNU |
|  | Run Length Non-Uniformity (RLNU) | GLRLM_RLNU |
|  | Run Percentage (RP) | GLRLM_RP |
| Textural features based on the Neighborhood Gray-level Different Matrix (NGLDM) | | |
|  | Coarseness | NGLDM_Coarseness |
|  | Contrast | NGLDM_Contrast |
|  | Busyness | NGLDM_Busyness |
| Textural features based on the Gray-Level Zone Length Matrix (GLZLM) | | |
|  | Short-Zone Emphasis (SZE) | GLZLM_SZE |
|  | Long-Zone Emphasis (LZE) | GLZLM_LZE |
|  | Low Gray-level Zone Emphasis ( LGZE) | GLZLM_LGZE |
|  | High Gray-level Zone Emphasis (HGZE) | GLZLM_HGZE |
|  | Short-Zone Low Gray-level Emphasis (SZLGE) | GLZLM_SZLGE |
|  | Short-Zone High Gray-level Emphasis (SZHGE) | GLZLM_SZHGE |
|  | Long-Zone Low Gray-level Emphasis (LZLGE) | GLZLM_LZLGE |
|  | Long-Zone High Gray-level Emphasis (LZHGE) | GLZLM_LZHGE |
|  | Gray-level Non-Uniformity (GLNU) | GLZLM_GLNU |
|  | Zone Length Non-Uniformity (ZLNU) | GLZLM_ZLNU |
|  | Zone Percentage (ZP) | GLZLM_ZP |
